# Communicating evidence about the environment’s role in obesity and support for government policies to tackle obesity: a systematic review with meta-analysis

**DOI:** 10.1080/17437199.2020.1829980

**Published:** 2020-10-02

**Authors:** James P. Reynolds, Milica Vasiljevic, Mark Pilling, Theresa M. Marteau

**Affiliations:** a Behaviour and Health Research Unit, University of Cambridge, Cambridge, UK; b Department of Psychology, Upper Mountjoy, Durham University, Durham, UK

**Keywords:** Causal beliefs, attribution, obesity, policy, attitudes

## Abstract

Public support for many policies that tackle obesity by changing environments is low. This may reflect commonly held causal beliefs about obesity, namely that it is due to failures of self-control rather than environmental influences. Several studies have sought to increase public support by changing these and similar causal beliefs, with mixed results. The current review is the first systematic synthesis of these studies. Searches of PsycInfo, Medline, Web of Science, Scopus, and Open Grey yielded 20 eligible studies (*N* = 8977) from 11,776 abstracts. Eligible studies were controlled experiments with an intervention group that communicated information about the environment’s role in obesity, and a measure of support for environment-based obesity policies. The protocol was prospectively registered on PROSPERO. Meta-analyses showed no evidence that communicating information about the environment’s influence on obesity changed policy support or the belief that the environment influences obesity. A likely explanation for this null effect is the ineffectiveness of interventions that were designed to change the belief that the environment influences obesity. The possibility remains, however, that the association observed between beliefs about the causes of obesity and attitudes towards obesity policies is correlational and not causal.

## Introduction

Scientific and policy communities now widely acknowledge that environmental factors are the driving force behind the high and rising rates of obesity worldwide (Brandkvist et al., 2019; Davies, 2019; Swinburn et al., 2011; Tyrrell et al., 2017). Reducing the health and economic burden of obesity would be helped by large scale changes to our physical and fiscal environments that enable healthier food consumption and increased physical activity (Hollands et al., 2019; Marteau et al., 2012). Changes such as reducing the purchasing of unhealthy foods by use of taxes, restrictions on advertising, restrictions on size, and restrictions on availability would make progress towards creating healthier food environments (Hollands et al., 2015; Hollands et al., 2019; Niebylski et al., 2015; Pechey et al., 2019; Veerman et al., 2009). Despite the growing evidence for the effectiveness of these interventions there has been mixed success globally in the implementation of these and similar interventions. This *Policy Inertia* has been attributed to three sets of factors: inadequate political leadership and governance; strong opposition to policies by powerful commercial interests; and lack of public demand or support for effective policies (Swinburn et al., 2019). The focus of the current review is on public support for these policies (Cullerton et al., 2016, 2018; Jones et al., 2016).

Public support tends to be higher for policies that are least effective (Diepeveen et al., 2013). This includes information-based strategies that have little or no effect at creating meaningful behaviour change over sustained periods of time (Van Sluijs et al., 2007; Wakefield et al., 2010). In contrast, taxes on unhealthy food and drink reduce consumption of the target product at scale (Niebylski et al., 2015; The Task Force on Fiscal Policy for Health, 2019) yet support for these approaches is typically lower than 50% (Eykelenboom et al., 2019).

Attribution Theory provides a framework for understanding varied support for different obesity policies (Heider, 1958). This theory addresses how people answer such questions as why are people poor, why people commit crimes, and why people are affected by obesity. People’s answers to these questions reflect their beliefs about the causes of these phenomena, which reliably predict their attitudes towards those groups, and towards policies that target aim to help those groups. For example, those who attribute obesity to the environment, tend to have the highest support for policies to reduce obesity (Hilbert et al., 2007; Petrescu et al., 2016; Reynolds et al., 2018). This is problematic as across numerous countries, people are more likely to attribute obesity to failures of self-control than to environmental forces (Mazzocchi et al., 2015; Reynolds et al., 2018).

Public health advocates have attempted to galvanise support for obesity policies by including messages that highlight the influence of food environments on excessive food intake that in turn influences obesity (Elliott-Green et al., 2016). While correlational evidence supports this approach, the experimental evidence provides mixed support. Several studies appear to suggest that communicating evidence of the environment’s influence on obesity has no effect on support for obesity policies (McGlynn & McGlone, 2018; Reynolds, Vasiljevic, et al., 2020; Young et al., 2016). Other studies report some statistically significant effects (Ortiz et al., 2016; Pearl & Lebowitz, 2014), but sometimes just in one subgroup (Garbarino et al., 2018). While these studies differ somewhat in the messages that they communicated to participants, this seems unlikely to account for these mixed effects given that first, there is a large amount of overlap in message content and second, there are conflicting results between studies using the same messages (Ortiz et al., 2016; Pearl & Lebowitz, 2014; Reynolds, Vasiljevic, et al., 2020). This warrants a systematic synthesis of the extant literature.

The messages evaluated in these studies rest on the assumption that the causal messages they contain – that environmental cues lead to excessive food consumption and thereby influences obesity – will change causal beliefs about obesity; which only one study has achieved (Ortiz et al., 2016). Changing beliefs about the other influences on obesity – such as attempting to weaken the belief that a lack willpower influences obesity – may be irrelevant. This is due to correlational analyses which show that attributing obesity to willpower, or even to genetics, has negligible or small positive associations with support for policies that aim to reduce obesity (Beeken & Wardle, 2013; Petrescu et al., 2016; Reynolds et al., 2018; Reynolds, Vasiljevic, et al., 2020). The hypothesis implicit in many of these studies is that any meaningful increase in support follows strengthening the belief that environmental cues influence obesity regardless of changes in beliefs about the role of willpower or genetics.

**Figure 1.**
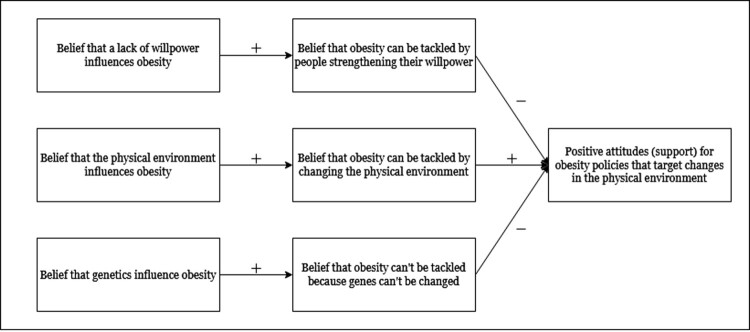
A conceptual model of the hypothesised relationships between the key constructs.

The primary aim of the current review is to investigate whether communicating information about environmental factors that influence obesity changes support for polices that aim to reduce obesity. Secondary aims are whether these messages also change beliefs about the role of the environment, genetics, and willpower in obesity. Beliefs about the role of genetics and willpower in obesity were examined to determine if the messages had any unintended effects. See Figure 1 for a conceptual model of the relationships between the constructs described here. To our knowledge, this is the first systematic review and meta-analysis addressing these questions.

## Method

This systematic review is reported in line with PRISMA (Preferred Reporting Items for Systematic Reviews and Meta-Analyses) guidelines (Moher et al., 2009). The review protocol was prospectively registered in the PROSPERO database (CRD: CRD42018099764). Data and code are available on the Open Science Framework (https://doi.org/10.17605/OSF.IO/ET49N).

### Eligibility criteria

*Studies*. Eligible studies were randomised experiments in which at least one group received information about the role of the obesogenic environment and at least one group was an eligible control or comparator.

*Interventions*. Eligible interventions were those that provided information or evidence that the obesogenic environment (e.g., the cost of foods, availability of unhealthy foods, lack of space for physical activity) is at least partly responsible for obesity. This included evidence presented in the form of results arising from research studies or statements from experts or recognised bodies (e.g., WHO). Also eligible were narratives describing an individual having difficulty losing weight due to the obesogenic environment in which they lived. Eligible interventions could be presented in any medium including text, infographic, audio, or video. An extract of an intervention from (Ortiz et al., 2016) is provided below which highlights influences of advertising and food placement:

Lately there has been a lot of talk about the factors that influence food choices in America. For example, food advertising can lead to the selection of unhealthy food and beverages. Certain food additives, such as extra salt, sugar, and caffeine, can also increase the desire for unhealthy food. And the placement of snack food and sugary beverages at checkout counters, especially in nongrocery retail stores, can often result in unintended food purchases and overeating. Consumers should be able to make their own dietary choices. But they also need to be free from the influence of heavy advertising, exposures to habit forming food ingredients, and invasive food product placement.

*Control groups*. Eligible control groups were those that received either no message or a message unrelated to the causes of obesity. For example, in one study (Niederdeppe et al., 2014) participants in the control group were provided with a text statement about a woman’s quest to discover a lost species of bird.

*Comparator groups*. Eligible comparator groups were those that received a message about the causes of obesity unrelated to food environments. This included messages describing the role of willpower or genetics in obesity.

*Participants*. There were no restrictions on participants.

*Primary outcome*. The primary outcome was the acceptability of a policy, defined as the level of support or attitude toward the implementation of that policy, using rating scales that allow a binary assessment (e.g., support or oppose) or a gradation of support or opposition such as seven point response scales. Ineligible measures of support were those that measured support for societal action in general and not linked to a specific action or policy (e.g., van der Linden et al., 2015). Also ineligible were indirect measures of support such as willingness to pay for a policy rather than actual support (e.g., Oliver & Lee, 2005).

*Secondary outcomes*. Secondary outcomes included beliefs about the causes of obesity including: (a) the belief that the obesogenic environment/society/food industry is responsible for obesity; (b) the belief that willpower/self-control/personal responsibility is responsible for obesity; and (c) the belief that genetics/biology/heredity is responsible for obesity.

*Policies*. Eligible policies were those that aimed to tackle obesity by changing cues in the physical environment (e.g., changing the availability of healthier food outlets, restricting marketing of unhealthy foods, changing the availability of larger portion sizes, or providing facilities for outdoor physical activity) or the economic environment (e.g., taxes on unhealthier foods, subsidies on healthier foods). Ineligible policies included those that were unrelated to obesity, or were not aimed at changing the environment to tackle obesity, such as those that aimed to reduce prejudice, offer legal protections, or punish people with obesity such as by increasing insurance premiums.

### Literature search

The search strategy was developed with the assistance of an information scientist. Four electronic databases of published studies were searched: PsycInfo, Medline, Web of Science, and Scopus, and one grey literature database: Open Grey. There were no restrictions made on publication date. The search terms were as follows:

(acceptab* OR support* OR attitude* OR oppose* OR opinion* OR favour*) AND (policy OR policies OR intervention* OR treatment* OR prevent* OR propos*) AND (attribution OR caus* OR “fundamental attribution error” OR “correspondence bias” OR environment* OR context OR situation) AND (obesity OR obese OR overweight OR weight) AND (experiment* OR “random allocation” OR “randomly allocated” OR “randomly assigned” OR “allocated randomly” OR “assigned randomly” OR "randomised controlled trial" OR RCT)

Database searches were completed up to September 2018. Title-abstract records were divided between two researchers and screened by one researcher only, with the advice to be lenient towards inclusion if unsure (Higgins & Green, 2008). Full texts of potentially eligible articles were screened by two researchers working independently. Any discrepancies were resolved by discussion or with a third researcher arbitrating as appropriate. Database searches were supplemented with forward and backward citation tracking (using Google Scholar) of eligible articles and contacting authors of eligible papers to request any further relevant studies or unpublished data.

### Data extraction

*Information extracted*. All information was extracted in duplicate with discrepancies resolved by discussion or with a third researcher arbitrating as appropriate.

*Coding*. A coding scheme was developed prior to data extraction. Five features of the interventions were coded: other information included in the interventions (information about the nature/magnitude/consequences of obesity; information about the effectiveness of the policies to tackle obesity; further information as described by the primary study’s authors), length of the intervention, readability of the intervention (as assessed by the Gunning Fog index), source of the information (experts, studies/research, member of the public, organisation), and medium (text, image, audio, video).

*Missing data*. There were 11 primary studies that did not report means, standard deviations, or sample sizes per group, which were the primary data that we used to conduct meta-analyses. Requests for data were sent to the corresponding authors from these 11 studies. Data were received for six of these studies. Most effect sizes were calculated from reported means, standard deviations, and sample size information. When these data were not available effect sizes were calculated from *F* statistics and sample sizes or odds ratios and sample size, using formulas available in Borenstein et al. (2011) or Wilson (n.d.). For the primary meta-analysis on policy support, there were 3/15 eligible studies for which we were unable to estimate effect sizes.

### Risk of bias

The Quality Assessment Tool for Quantitative Studies (Effective Public Health Practice Project, 1998) was used to assess the quality of each study based on: selection bias, study design, confounders, blinding, data collection methods, and withdrawals and dropouts. An overall quality score was derived from these categories: weak, moderate, or strong. The initial agreement between the two primary reviewers was moderate (linear weighted *κ* = .57) however all discrepancies were resolved by discussion or with a third researcher arbitrating as appropriate. Sensitivity analyses were conducted to determine if the main results were unchanged after only including studies that were not at high risk of bias. Funnel plots and Egger’s regression were used to assess small study bias (e.g., publication bias).

### Synthesis of results

Quantitative synthesis (meta-analysis) was used to calculate summary effect sizes using R v3.6.1. The primary meta-analyses examined the effect of communicating interventions compared to control groups. Two strategies were used to ensure independence of observations in each meta-analysis*: i.* in studies that included multiple eligible outcome measures, the combined means and variances were calculated based on guidance reported in Borenstein et al. (2011), taking the conservative approach of assuming correlations between variables were 1.0; and, *ii.* when multiple interventions were eligible, the least confounded intervention message was selected. For example, an intervention containing (1) information on the environment’s influence on obesity would be chosen over an intervention containing (1) this same information, and (2) information on the role of willpower in the development of obesity. Multiple eligible intervention groups were combined into a single group if no one intervention could be selected using this approach, as recommended in the Cochrane Handbook (Higgins & Green, 2008).

The secondary meta-analyses examined the effect of interventions communicating the environment’s influence on obesity against comparators, i.e., those that communicated information that obesity is caused by non-environmental factors such as genetics or willpower. All meta-analyses reported used random effects models and the effect sizes are reported in Hedge’s *g*.

An exploratory meta-regression was also conducted which aimed to examine whether greater changes in the belief that the environment causes obesity were associated with greater changes in public support for obesity policies.

## Results

### Study selection

**Figure 2.**
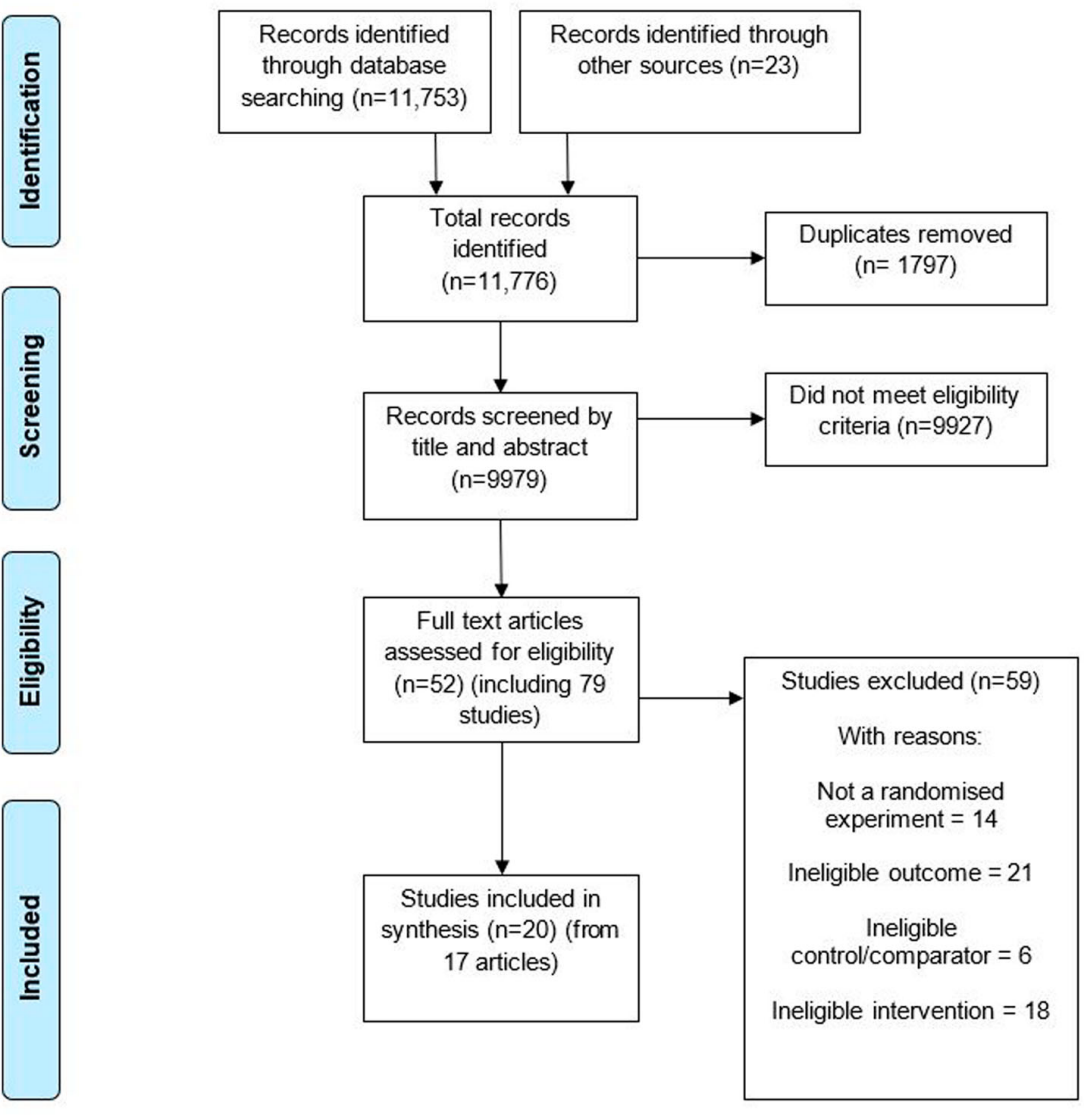
PRISMA flow chart displaying study flow.

Figure 2 displays the flow of studies through the systematic review process. 9979 study records were screened based on their titles and abstracts. Full-text screening of 52 articles that were judged to be potentially eligible resulted in 20 eligible studies from 17 articles (*N* = 8977 participants). Of these, 12 studies (*N* = 7353) were analysed in the primary analysis testing the impact of the interventions *vs.* control groups on support for obesity policies.

### Study characteristics

There were 20 eligible studies in current review. The majority were conducted in the USA (85%), with the others in the UK (10%), and South Korea (5%). The majority used adults sampled from the general population (80%), with others recruiting university students (5%), parents of elementary school children (5%), state legislators (5%), or adults with obesity or an unhealthy BMI (5%). The interventions were mostly communicated via text (55%), followed by text + image(s) (35%), text + infographics (%), and text + audio (5%). Most of the included studies had one control group (60%), some had no control group but one or more comparator groups (25%), and some included both a control group and one or more comparator groups (15%). There were a wide range of eligible policies to tackle obesity and all studies assessed support for multiple policies (range: 3–12 policies). Items measuring support for policies were typically combined into one or more outcome measures. Examples of policies include: prohibit advertising of unhealthy foods high in fat and sugar in schools; warning labels on foods with high sugar, eliminate fast-food from schools; have zoning laws requiring that all new residential and commercial developments include sidewalks and other safe paths to encourage physical activity; tax on sugar-sweetened beverages; limit on the size of sugar-sweetened beverages sold in restaurants; calorie labels on restaurant menus; a policy to increase the availability of healthy foods in worksites, schools, and hospitals; and provide grants to independent grocery stores to sell healthy products in locations where supermarkets are not available. Further details can be found in the summary of studies table (see Table S1).

The studies were funded by the Robert Wood Johnson Foundation (35%), departments within individual universities (15%), and the National Institute for Health Research (15%). The authors of one study declared that the study was not funded. The remaining (30%) studies did not report the source of their funding.

### Risk of bias within studies

The modal quality score was *low quality*, with nine out of 20 studies being thus scored. Five were rated *moderate quality* and six were deemed *high quality*. The most common reason for lower scores was studies recruiting non-representative samples.

### Risk of bias across studies

**Figure 3.**
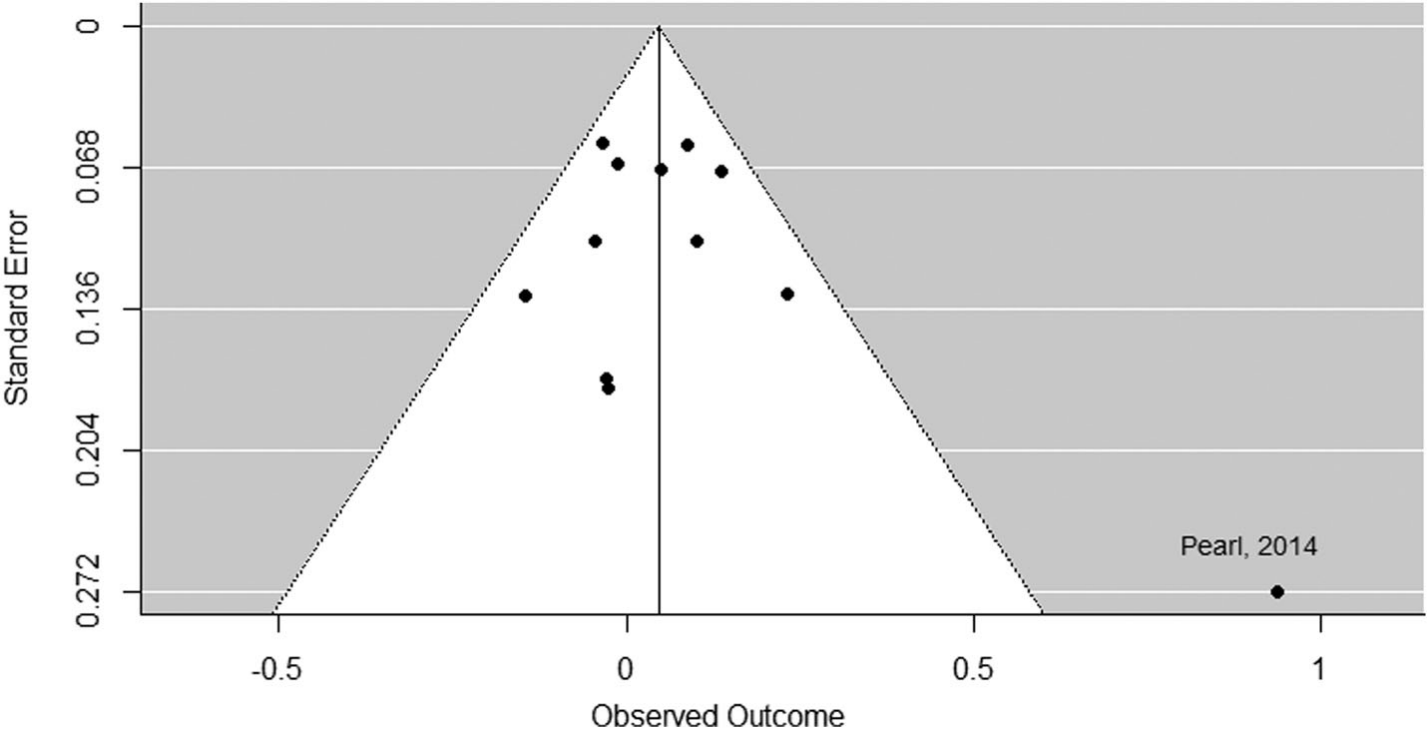
Funnel plot: Information about the environment’s role in obesity vs. no message control group on support for obesity-related policies.

**Figure 4.**
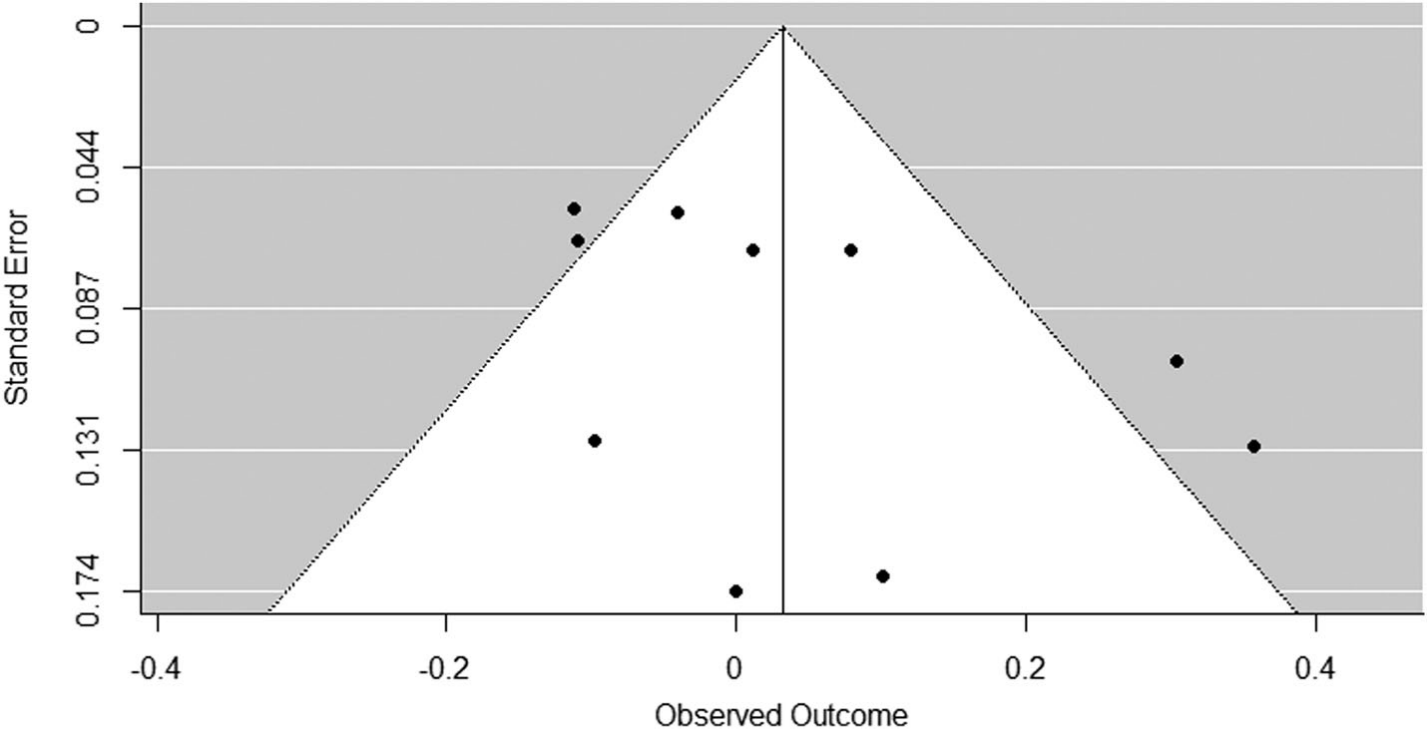
Funnel plot: Information about the environment’s role in obesity vs. a no message control group on beliefs about the environment’s influence on obesity.

There was no evidence of small study bias across the studies. Examination of the funnel plots and Egger’s regressions suggested no asymmetry for the policy support outcome, *z* = 1.50, *p* = .132 (see Figure 3), for the causal beliefs: environment outcome, *z* = 1.48, *p* = .138 (see Figure 4), for the causal beliefs: genetics outcome, *z* = 1.19, *p* = .235 (see Figure S2), or for the causal beliefs: willpower outcome, *z* = 0.12, *p* = .907 (see Figure S4).

### Main results

#### Policy support

**Figure 5.**
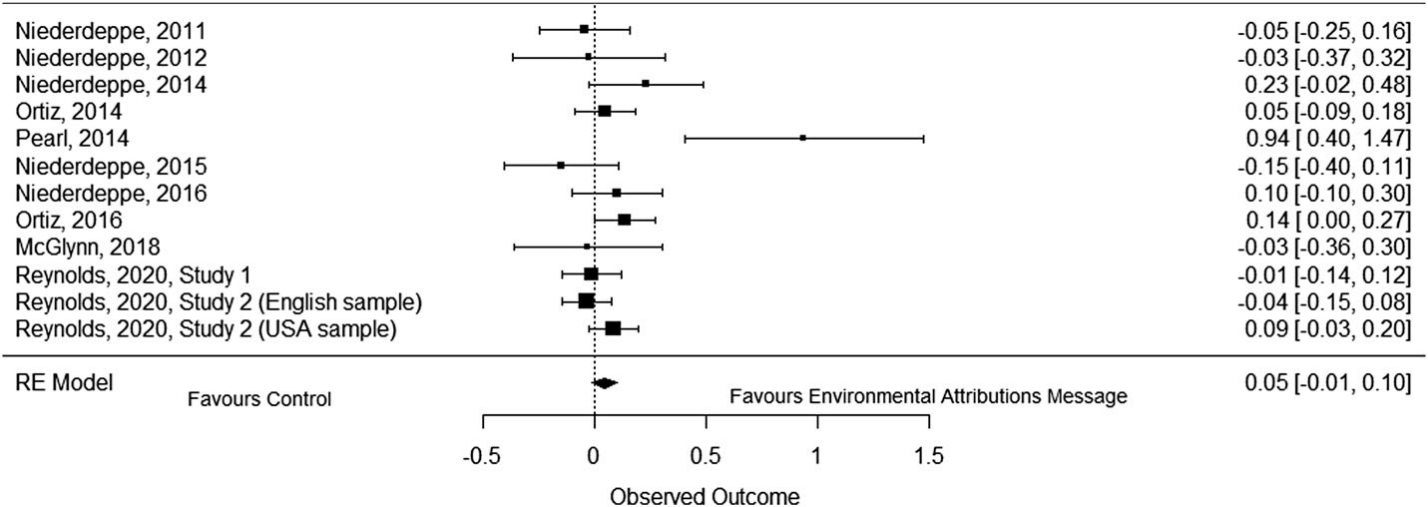
Forest plot of comparison: Information about the environment’s role in obesity vs. no message control group on support for obesity-related policies.

Communicating information that the environment influences obesity had no meaningful effect on support for obesity policies when compared to a control group, *g* = .05, 95% CI [−.01, .10], *p* = .105, *k* = 12 (see Figure 5). There was some evidence that low heterogeneity was present, *Q*(11) = 21.52, *p* = .028, *I^2^* = 17%, *τ*^2^ = .002, *τ* = .04, suggesting that the results vary more than would be expected by chance alone. Removing an outlier (Pearl & Lebowitz, 2014) from this analysis did not substantively change the results, *g* = .04, 95% CI [-.02, .09], *p* = .171, *k* = 11, but reduced some of the heterogeneity between studies, *Q*(10) = 10.62, *p* = .388, *I^2^* = 10%, *τ*^2^ = .001, *τ* = .03.

Excluding studies that were rated as low quality also had no substantive effect on the results, *g* = .04, 95% CI [-.02, .11], *p* = .192, *k* = 7. This suggests that the main results are robust to variations in study quality.

#### Causal beliefs: environment

**Figure 6.**
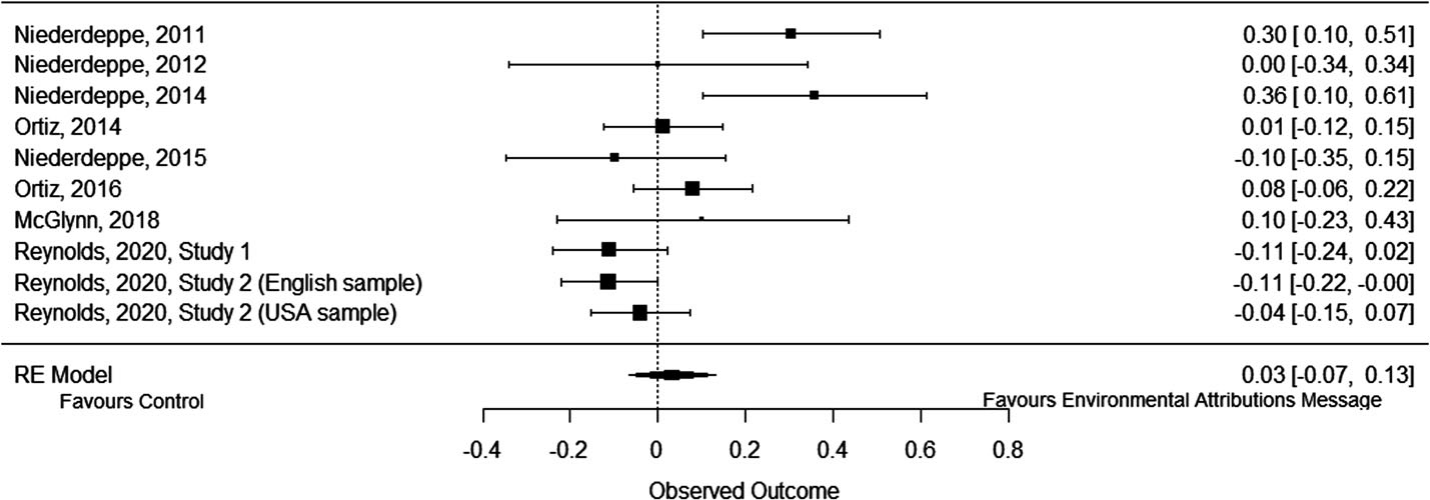
Forest plot of comparison: Information about the environment’s role in obesity vs. a no message control group on beliefs about the environment’s influence on obesity.

Communicating information that the environment influences obesity had no meaningful effect on the belief that the environment influences obesity when compared to a control group, *g* = .03, 95% CI [−.07, .13], *p* = .524, *k* = 10 (see Figure 6). There was significant and substantial heterogeneity, *Q*(9) = 25.67, *p* = .002, *I^2^* = 70%, *τ*^2^ = .02, *τ* = .13. This suggests that the results vary more than would be expected by chance alone.

Excluding studies that were rated as low quality did not substantively change the results, *g* = −.01, 95% CI [−.10, .08], *p* = .853, *k* = 7. This suggests that the main results are robust to variations in study quality.

### Further analyses

The secondary analyses investigating the effect of the interventions on beliefs about the role of genetics and willpower in obesity are reported in the supplement.

Further meta-analyses were run that compared interventions communicating the influence of environments on obesity against comparator messages for each of the four outcome measures. These are also reported in the supplement. The inclusion of information about a range of causes of obesity in the comparator messages makes any inferences about the impact of the interventions unreliable and there were too few studies to run subgroup analyses to explore this further.

### Exploratory analyses

An exploratory meta-regression was conducted to assess whether changes in the belief that the environment influences obesity engenders changes in support for obesity policies. In this regression model, *y* = Hedge’s *g* (outcome: policy support; comparison: intervention *vs.* control) and *x* = Hedge’s *g* (outcome: belief that the environmental influence obesity; comparison: intervention *vs.* control).

The results suggested a positive, but statistically non-significant relationship between changes in belief that the environment causes obesity and changes in support for obesity policies, *B* = .29, 95% CI [−.17, .75], *p* = .181.

## Discussion

The results of this systematic review provide no evidence that communicating information about the role of the environment on obesity leads to changes either in support for obesity policies to change the environment or beliefs about the influence of the environment on obesity. As this latter belief did not change following exposure to the intervention messages, it remains possible that changing this belief would change public support. An exploratory meta-regression analysis suggested a positive albeit non-significant relationship between belief change and policy support change. Therefore, there is no evidence that the messages which were more persuasive at changing causal beliefs were more successful at changing attitudes. The possibility remains that the association observed between beliefs about the causes of obesity and attitudes towards obesity policies is correlational and not causal.

Two of the 20 included studies provided evidence that the intervention message increased support for obesity policies as a main effect – i.e., across the whole study sample (Ortiz et al., 2016; Pearl & Lebowitz, 2014). Although the interventions mostly differed across studies, there are two principle reasons why intervention content is unlikely to fully account for these discrepant results. First, interventions sourced from these two studies were used in a third study that did not replicate the results (Reynolds, Vasiljevic, et al., 2020). Second, the intervention messages across all studies contained largely overlapping information. It is possible that different intervention content does vary the effectiveness of the message to some extent but, due to the large variation in effect sizes, it is unlikely to fully account for the discrepant results. Although it is not clear why these discrepant results occurred, a further study ruled out the possibility that this was due to two characteristics of participants across studies: country of residence and BMI (Reynolds, Vasiljevic, et al., 2020).

Secondary meta-analyses showed that the interventions did not change beliefs about the roles of the environment and genetics in obesity, but they did strengthen the belief that willpower influences obesity. This effect is at odds with the content of the messages which did not aim to target this specific belief. This may be a back-fire effect in which participants react to information with which they disagree by strengthening their initial causal beliefs to the neglect of new evidence (e.g., Nyhan & Reifler, 2010). As no studies in the current review measured causal beliefs before and after intervention messages, it is not possible to test this hypothesis, i.e.*,* that pre-existing causal beliefs about obesity moderate the effect of messages about these causes. Despite the current review finding no evidence that the target belief – that the environment influences obesity – was changed by messages designed to change it, the increase in the belief that willpower causes obesity was not accompanied by a statistically significant decrease (or increase) in policy support. Numerous correlational analyses also show that attributing obesity to failures of willpower has at best negligible associations with policy support and not negative associations (Beeken & Wardle, 2013; Petrescu et al., 2016; Reynolds et al., 2018; Reynolds, Vasiljevic, et al., 2020). It may then not matter if causal beliefs about willpower are changed provided the belief that the environment causes obesity is strengthened.

Even if the proximal causal belief is changed, the causal chain that leads from changes in beliefs to support for policy is unclear. Some of the studies included in this review shed some light on possibilities. For example, if people believe that certain aspects of the environment cause obesity, then they may ascribe responsibility to change these aspects of the environment to certain groups such as governments or business (Jeong et al., 2018; Niederdeppe et al., 2011). A further route through which causal beliefs may affect attitudes is the affective component of attitudes. For example, if a person is perceived to have obesity due to environmental causes then others may feel greater empathy for their situation, which may lead to greater desire for government to help such individuals (Niederdeppe et al., 2015). The attribution-value model of prejudice also predicts that attributions alone are not sufficient in predicting attitudes, but that they depend on cultural values (Crandall et al., 2001). This was shown in samples from six countries, in which attributing obesity to personal responsibility and having a negative cultural value toward obesity improved predictions of prejudice above the individual effects of these two variables. Testing these different hypothetical causal chains is difficult without successfully changing the belief that the environment causes obesity, and the current meta-analysis found no evidence that many different messages were sufficiently persuasive to accomplish this.

### Limitations and future directions

This systematic review with meta-analysis provides the first synthesis of evidence for the role of causal beliefs about obesity and attitudes toward obesity policies. The results are also robust given the use of Cochrane methods for the review.

Several limitations should, however, be noted. Although there was no evidence of a small study bias for the primary analysis, there was one obvious outlier visible from observing the funnel plot and the forest plot (Pearl & Lebowitz, 2014). After the analysis was re-run without this study, the findings were unchanged. There was evidence of heterogeneity in the secondary meta-analysis that examined the effect of the interventions on beliefs about the environment’s influence on obesity. This suggests that the results vary more than would be expected by chance and indicates the presence of effect moderators, however it is not clear what these may be. One possible explanation is that different studies measured beliefs about different aspects of the environment, including: food advertising, the food industry, lack of safe and affordable places to exercise, the high availability of unhealthy foods, the high cost of healthy food, and the low cost of unhealthy food. Despite the high heterogeneity for this secondary outcome, the heterogeneity was low for the primary outcome, suggesting that there is consistently no effect of communicating information about the environment’s influence on obesity on policy support.

There were 15 eligible studies for the primary analysis however we were only able to estimate effect sizes for 12 of these due to incomplete reporting of statistics. The description of the results in one these studies with incomplete data reports mixed results (Zhou & Niederdeppe, 2017), and the two remaining studies support the conclusions reached in the current meta-analysis: that there is no effect of these messages on policy support (Barry et al., 2013; Husmann, 2015).

The quality assessment process suggested that although the mode quality score was low (45%), the majority of studies were either of moderate or high quality (55%). The main reason for lower quality scores was the recruitment of non-representative samples and failure to report primary outcomes that had received validity testing which should be addressed in future research. This potentially could suggest that the results may not generalise to wider populations and that the outcome may not be measuring what the authors intended. Against this, the results were robust to variations in quality as sensitivity analyses showed similar results to the original results after excluding low quality studies. While this suggests that the results are robust, there are further concerns about the generalisability of the results. The majority of studies (19/20) took place in either the USA or the UK. Further research needs to test these hypotheses amongst non-WEIRD samples, i.e., those who are not just from Western, Educated, Industrialised, Rich, and Democratic countries (Henrich et al., 2010).

This review identified the failure of intervention messages to change target causal beliefs. Some of these messages did include established persuasive techniques such as the use of images to highlight the key message (Miniard et al., 1991), citing an authority as the origin of any evidence (Petty & Cacioppo, 1986; Pornpitakpan, 2004), and combining both evidence and individual narratives. These and techniques from other fields could be used to improve the effectiveness of intervention messages. For example, reporting that there is a scientific consensus on the causes of obesity (van der Linden et al., 2015), repeating the key message multiple times (Cacioppo & Petty, 1979), or using video as a medium to communicate the message (Goldberg et al., 2019; Luecking et al., 2017). While these techniques offer promise, there is evidence that support for policies is difficult to change via altering characteristics of messages (Reynolds et al., 2019; Reynolds, Stautz, et al., 2020).

## Conclusions

The current systematic review with meta-analysis found no evidence that communicating information on the role of the environment in obesity can increase support for obesity policies or change beliefs about the influence of the environment on obesity. This is likely due to the insufficiently persuasive messages that were used in the primary studies and it remains possible that if new approaches change the belief that the environment causes obesity, then support for obesity policies may increase. However, based on the presently available evidence, it cannot be concluded that causal beliefs are important in shaping or forming attitudes – at least within the context of obesity policies – and therefore targeting causal beliefs does not currently represent a viable strategy for increasing public support.
